# Breast Cancer Metastases to the Gastrointestinal Tract Presenting with Anemia and Intra-abdominal Bleed

**DOI:** 10.7759/cureus.1429

**Published:** 2017-07-06

**Authors:** Idrees Khan, Rehan Malik, Amina Khan, Salman Assad, Mehr Zahid, Muhammad Saad Sohail, Faizan Yasin, Ahmed H Qavi

**Affiliations:** 1 Center for Healthcare Advancement & Outcomes Research, Baptist Health Medical Group; 2 Internal Medicine, Mount Sinai Medical Center, Miami Beach, Florida,; 3 Shifa Tameer E Millat University, Shifa International Hospital, Islamabad, Pakistan; 4 Department of Medicine, Shifa International Hospital, Islamabad, Pakistan; 5 Internal Medicine, University of Lahore, Lahore, Pakistan; 6 Shifa International Hospital, Shifa International Hospital, Islamabad, Pakistan; 7 Department of Medicine, Shifa Tameer-e-Millat University; 8 Department of Medicine, Mahroof Hospital

**Keywords:** breast cancer, metastasis, case reports, gastrointestinal bleed, anemia

## Abstract

Signet ring adenocarcinoma of the breast with synchronous metastasis to the gastrointestinal (GI) tract is a rare occurrence, typically presenting with abdominal pain, dyspepsia, or GI bleed. We report a case of metastatic breast cancer presenting with a complaint of anemia. A further diagnostic evaluation revealed generalized lymphadenopathy, nodular thickening of the urinary bladder wall, bone lesions, and enlarged pancreas. Biopsies from the lymph nodes, pancreatic biopsy, and bladder nodule all revealed a signet cell carcinoma. An upper and lower GI endoscopy revealed multiple ulcerated gastric mucosal nodules and polypoid folds in the cecum and proximal ascending colon; the biopsies from these lesions were also positive for signet ring cell adenocarcinoma.

## Introduction

Breast cancer is the most documented malignancy in women and an important factor leading towards the morbidity and mortality in women aged 20-59 years [1]. It accounts for more than 25% of all newly diagnosed cancers in females and is responsible for 15% of the cancer-related deaths in women [1]. The metastatic disease usually develops in 75% of breast cancer patients with or without treatment of the primary malignancy. This metastatic behavior of breast cancer, once it penetrates through the gastrointestinal (GI) tract, usually involves the upper GI tract such as the stomach, small bowel, and pancreaticobiliary regions. This is a very rare form of metastasis [2]. Signet ring adenocarcinoma of the breast with synchronous metastasis to the GI tract is a rare finding presenting with abdominal pain, dyspepsia, or GI bleed. We report an occult breast cancer presenting with severe anemia and synchronous metastasis to the GI tract.

## Case presentation

**Figure 1:**
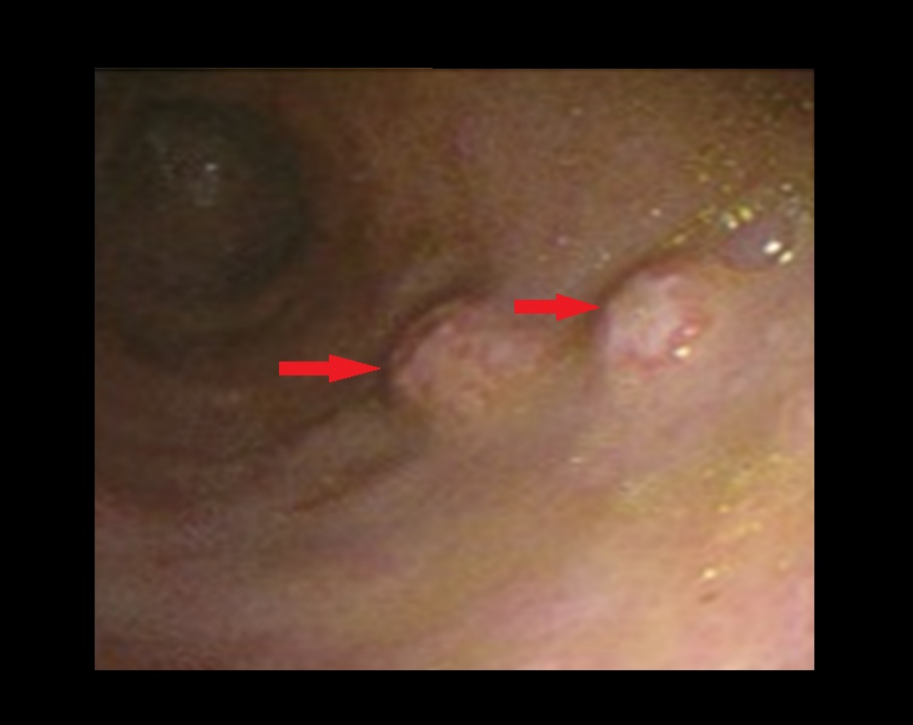
Esophagogastroduodenoscopy (EGD) EGD showing ulcerated nodular lesions (red arrows).

**Figure 2:**
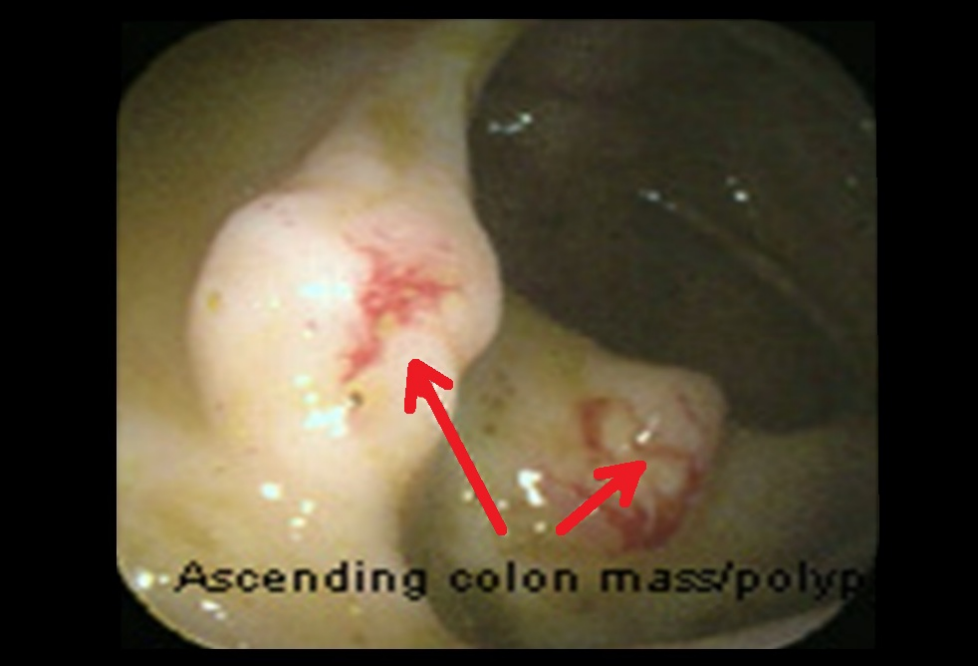
Colonoscopy Colonoscopy showing ulcerated polypoid nodules (red arrows).

A 56-year-old woman, with a history of hypertension and hypothyroidism, presented with progressive weakness and was found to be severely anemic with a hemoglobin of 5.2 gm/dl, low mean corpuscular volume (MCV), and low iron saturation. A subsequent workup revealed generalized lymphadenopathy, bone lesions, enlarged pancreas, and nodular thickening of the urinary bladder wall. Biopsies from the lymph nodes, pancreatic biopsy, and bladder nodule all revealed a signet cell carcinoma. An upper and lower GI endoscopy revealed multiple ulcerated gastric mucosal nodules and polypoid folds in the cecum and proximal ascending colon; the biopsies from these lesions were also positive for signet ring cell adenocarcinoma (Figures 1-2). Deoxyribonucleic acid (DNA) microarray testing of the lymph node biopsy showed that the primary tissue of origin was consistent with the breast. The patient was started on chemotherapy, yet her condition deteriorated, and she died. An autopsy revealed a right breast cancer with extensive widespread metastases including the stomach, small intestine, and colon.

 

## Discussion

Breast cancer is the most common cancer among women, but it is only second to melanoma as the most frequent primary with metastases to the GI tract. Such metastases occur only in 4%-18% of patients, especially in lobular carcinoma [1]. The most commonly involved part is the stomach. When breast carcinoma metastasizes to the stomach, it is important to differentiate it from a primary gastric carcinoma as the treatment plan for these two malignancies are different. This can be done on the basis of clinical, endoscopic, radiological, and histopathological features [2].

Patients with GI metastasis can present with abdominal pain, nausea, vomiting, dysphagia, early satiety, weight loss, tenesmus, or gross bleed. Presentation with severe symptomatic iron deficiency anemia is very unusual and to our knowledge, has rarely been reported. The signet ring cell feature is characteristic of not only gastric but also of other GI adenocarcinoma, and this feature can add to the diagnostic dilemma in such cases [3]. The availability of DNA microarray has facilitated a distinction. The degree of anemia signals advanced disease and a long history of GI involvement.

Invasive ductal carcinoma (IDC) typically metastasizes to the lung, liver, or bone whereas Invasive lobular carcinoma (ILC) more commonly metastasizes to the GI tract and sometimes to the peritoneum and retroperitoneum [3]. This behavior has been explained by various studies, most of which state that this is due to the small size and shape of ILC cells, with E-cadherin overexpression favoring dis-cohesiveness between the cells to migrate to areas of microanatomy that are more conducive to stopping these cells [4]. The difference in metastatic behavior of ILC can be explained by the survival and growth factors provided by the ovaries and peritoneum [5]. Gastric metastasis alone, colon metastasis alone, and metastases in both locations from the breast cancer have been described, but, to our knowledge, there haven’t been too many cases with simultaneous spread to the stomach and colon presenting with severe anemia as the first manifestation of the disease [6-8].

One such case was reported by Villa Guzman, et al. where the patient presented with severe dyspeptic symptoms like nausea and epigastric pain. She underwent an esophagogastroscopy that showed signs of malignancy; biopsies were taken from the suspicious gastric and colonic areas which revealed metastases from lobular carcinoma of the breast [9].

Although IDC and ILC are the more common types of breast carcinoma, there has been a case of phyllodes tumor that was reported recently. The presentation was quite similar to our case. This was a 44-year-old patient who presented with similar symptoms of anemia (hemoglobin levels of 6.7 g/dL) and subsequently, an esophagogastroduodenoscopic (EGD) evaluation was performed, which identified a large gastric mass (approximately 7 cm in diameter) with active bleeding and other related masses. In addition to that, the same study points out the importance of considering GI metastasis as the cause when any patient with breast carcinoma presents with severe anemia, followed by proper workup in order to rule out any bleeding foci. This is also important when considering chemotherapy or targeted therapy as some of these can increase the risk of bleeding [10].

## Conclusions

Signet ring adenocarcinoma of the breast with metastasis to the GI tract is a rare entity. Occult breast cancer with synchronous metastases to the stomach and intestine and severe microcytic anemia is an unusual presentation and can delay the diagnosis. Therefore, an in-time diagnosis could have led to an early treatment that would have increased the rate of survival.
